# Avatar generator: a tool for prediction of three-dimensional humanoid avatars from manual anthropometric measurements

**DOI:** 10.3389/fnut.2026.1906710

**Published:** 2026-09-15

**Authors:** Marco Alessandro Minetto, Marta Boccardo, Antonio Cappuccio, Marta Andrighetti, Chiara Busso, Stefano Basso, John A. Shepherd, Steven B. Heymsfield, Angelo Pietrobelli, Alberto Botter

**Affiliations:** 1 Division of Physical Medicine and Rehabilitation, Department of Surgical Sciences, University of Turin, Turin, Italy; 2 Laboratory for Engineering of the Neuromuscular System (LISiN), Department of Electronics and Telecommunication, Politecnico di Torino, Turin, Italy; 3 PoliToBIOMed Lab, Politecnico di Torino, Turin, Italy; 4 IT Systems, Website Management, E-Learning Division, University of Turin, Turin, Italy; 5 Department of Epidemiology, University of Hawaii Cancer Center, Honolulu, HI, United States; 6 Pennington Biomedical Research Center, Baton Rouge, LA, United States; 7 Pediatric Unit, Department of Surgical Sciences, Dentistry, Gynaecology and Pediatrics, University of Verona, Verona, Italy

**Keywords:** anthropometry, avatar, body circumferences, body lengths, optical scanning, total body volume

## Abstract

**Background:**

Three-dimensional avatars provide an intuitive visual representation of body size and shape that can be useful for tailored clinical care, for example in the management of persons with obesity. This study aimed to evaluate the hypothesis that avatars predicted from manual anthropometric measurements (i.e., synthetic avatars) are comparable to avatars obtained through a digital anthropometric approach (i.e., ground-truth avatars).

**Methods:**

A convenience sample of 83 adult participants was recruited: body weight and height were measured and anthropometric assessments were performed in each participant. The manual measurements included nine circumferences (neck, mid-upper arm, wrist, chest, natural waist, belly button waist, maximal hip, thigh, knee) and three lengths (shoulder width, upper limb length, inseam): a previously developed model was adopted to generate synthetic avatars from these twelve measurements (in combination with weight and height). The digital anthropometric assessment consisted of three-dimensional optical scanning. Synthetic and ground-truth avatars were digitally registered and reposed using Meshcapade.

**Results:**

Synthetic and ground-truth avatars had similar visual appearances and correlations between ground-truth and predicted measurements were all high (and statistically significant) with R^2^s ranging from 0.84 to 0.99 in males and from 0.77 to 0.99 in females. The lowest correlation was obtained for the thigh circumference in males and for the neck base circumference in females, while the highest correlation was obtained in both genders for the total body volume.

**Conclusion:**

Synthetic avatars predicted from manual anthropometric measurements were comparable to ground-truth avatars. The use of synthetic avatars can be useful in the clinical practice to support patient education and engagement in weight loss nutritional programs. To promote the clinical use of digital anthropometry, the avatar generator model presented in this study was made available as a simple user interface-based calculator (https://www.avatargenerator.unito.it/).

## 1 Introduction

Obesity has traditionally been defined using body mass index, but it is well-known that this anthropometric variable fails to fully capture important factors such as amount and distribution of body fat, metabolic health, and other variables that can contribute to the obesity-related health complications (1–4). The new clinical obesity definition emphasized the need to consider obesity on a spectrum from pre-clinical to clinical obesity, looking not only at the body mass index, but also at additional anthropometric measures such as the absolute or normalized (to hip circumference or to height) value of the waist circumference (5). The anthropometric evaluation in the clinical settings is commonly performed by tape-based manual assessment (6). However, smartphone camera technologies and optical body scanners became available during the last decade and can be now used to obtain a “virtual twin” (i.e., a three-dimensional avatar mesh of the human body) with automated anthropometry (6–8). Although multiple systems are already marketed to the sports and clothes fitting markets, their unavailability in most clinical settings and the apparent complexity of their use can represent barriers to the adoption of three-dimensional humanoid avatars (and digital anthropometric measurements) in the management of persons with obesity. An alternative approach for obtaining (and using in research studies and in clinical practice) humanoid avatars is to predict an individual three-dimensional representation from demographic (e.g., gender and age) and physical (e.g., height, weight, etc.) characteristics. For example, McCarthy et al. (9) have recently proposed and validated a manifold regression model to predict humanoid avatars from gender, age, weight, height and percent body fat (derived from dual-energy x-ray absorptiometry). Interestingly, this model was adopted by Ramirez et al. (10) for visual demonstration of tirzepatide-induced weight loss through the pre-post-treatment comparisons of avatars obtained from data (age, weight, height, and waist circumference) of participants in the Surmount 1 trial. Other models from predicting humanoid avatars from anthropometric measurements were previously proposed by Pujades et al. (11) and by Zeng et al. (12). The former model enables to predict humanoid avatars from height, weight, and simple anthropometric measurements (arm span, arm length, hip width) that can be acquired by the subject by using virtual reality game controllers, while the latter model enables to predict an avatar from up to nineteen measurements that can be acquired by conventional manual anthropometry. Although the latter model is user-friendly and can also be applied with a limited number of anthropometric parameters (i.e., in case of missing data), it was not validated in an independent sample of subjects. Therefore, the aim of our study was to evaluate the hypothesis that avatars predicted from an adapted implementation of this model on the basis of manual anthropometric measurements (i.e., synthetic avatars) are comparable to avatars obtained through digital anthropometric approach (i.e., ground-truth avatars).

## 2 Materials and methods

### 2.1 Participants and protocol

A convenience sample of 83 participants of both genders participated in the study. Healthy subjects and persons with obesity were included. Exclusion criteria were the presence of metabolic, neurological, or other severe medical conditions hindering the ability to participate in the study. Manual and digital anthropometric assessments were performed in all participants in a single experimental session. All subjects gave their written consent after receiving a detailed explanation of the protocol. The study conformed to the guidelines of the Declaration of Helsinki and was approved by the local ethics committee (protocol n. 0065654).

### 2.2 Body weight and height assessments

Body weight and height were assessed with each subject in undergarments and barefoot. Body weight and height were measured (to the nearest 0.1 kg and 0.5 cm, respectively) using a standard scale with a stadiometer (model Seca 799, Seca GmbH & Co., KG, Hamburg, Germany).

### 2.3 Digital anthropometric assessment

Optical images were taken with ProScanner device (version 5.0, Fit3D, Sacramento, CA, USA), using a standardized protocol, as previously described (13). Briefly, each participant was asked to stand upright in a standardized A-pose (with shoulders relaxed and arms positioned straight and abducted from the torso) while grasping the telescoping handles. A full body scan takes ∼45 s during which light-coding depth sensors capture the three-dimensional shape as the platform rotates once around. The acquisition of the three-dimensional shape was performed in duplicate to obtain two avatars for each participant. Each avatar consists of a mesh connected by triangles with approximately 300,000 vertices and 600,000 faces to represent body shape.

### 2.4 Manual anthropometric assessment

Manual anthropometric assessment was made on each participant by a single highly trained staff member using a calibrated flexible tape. The following circumferences were measured in duplicate according to the anatomical landmarks of the Blue Measurement guide (14) (reported in the Supplementary Table 1 and shown in the Supplementary Figures 1, 2) and the averages of two measurements for axial sites and four measurements for appendicular sites (two measurements for each of the two sides) were considered: neck, mid-upper arm, wrist, chest, natural waist, belly button waist, maximal hip, thigh, knee. Moreover, the following lengths were also measured in duplicate and the averages of two (for axial sites) or four (for appendicular sites) measurements were considered: shoulder width, upper limb length, inseam.

### 2.5 Human body reshaping model

The generation of synthetic avatars from manual anthropometric measurements was based on an adapted implementation of the three-dimensional human body reshaping model proposed by Zeng et al. (12). This model is based on a feature-selection-based local mapping technique and on a statistical representation of the human body that predicts the three-dimensional body shape from anthropometric measurements. The relationship between body measurements and body shape is learned from the CAESAR database of three-dimensional scans (a large dataset including over 4,000 individuals from the U.S., The Netherlands, and Italy) (15), and the input measurements are used to reconstruct an individualized three-dimensional body mesh that reflects the subject’s morphology.

In the present study, this framework was extended through a two-step refinement procedure aimed at improving the agreement between input anthropometry and reconstructed body shape. First, an initial avatar was generated from the anthropometric measurements provided by the user (including weight, height, and selected circumferences and lengths – see paragraph “2.3 Digital anthropometric assessment”) using the model proposed by Zeng et al. (12). The model is designed to use up to nineteen anthropometric parameters, and missing values are automatically estimated using a multivariate imputation by chained equations (MICE) algorithm. From this preliminary mesh, a set of anthropometric parameters was re-estimated directly on the reconstructed geometry, using the same anatomical control points that define the input parameters, so that input and re-estimated measurements remain directly comparable. In particular, body circumferences were computed through cross-sectional slicing of the mesh: the mesh was cut with an anatomically oriented plane at the corresponding control-point level and the convex hull of the resulting cross-section was calculated. Body weight was re-estimated from the mesh volume using the following gender-specific regression equations (11):

Male weight (kg) = [1,056.44 x volume (m^3^)] – 5.28
Female weight (kg) = [1,001.44 x volume (m^3^)] – 2.36

Linear measurements not directly recomputed at this step (e.g., shoulder width, upper limb length, inseam) were retained from the initial parameter set. Afterward, a measurement compensation step was applied to reduce the reconstruction errors. For each available anthropometric parameter, a correction factor was computed based on the ratio between the original input measurement and the corresponding value extracted from the initial avatar. These compensated measurements were then used as input for a second iteration of the reshaping model. Finally, a refined avatar was generated using the compensated parameter set. Each avatar consists of a mesh connected by triangles with approximately 18,500 vertices and 37,000 faces to represent body shape. A schematic overview of the full procedure, from the manual input measurements to the final compensated avatar, is provided in Figure 1.

**FIGURE 1:**
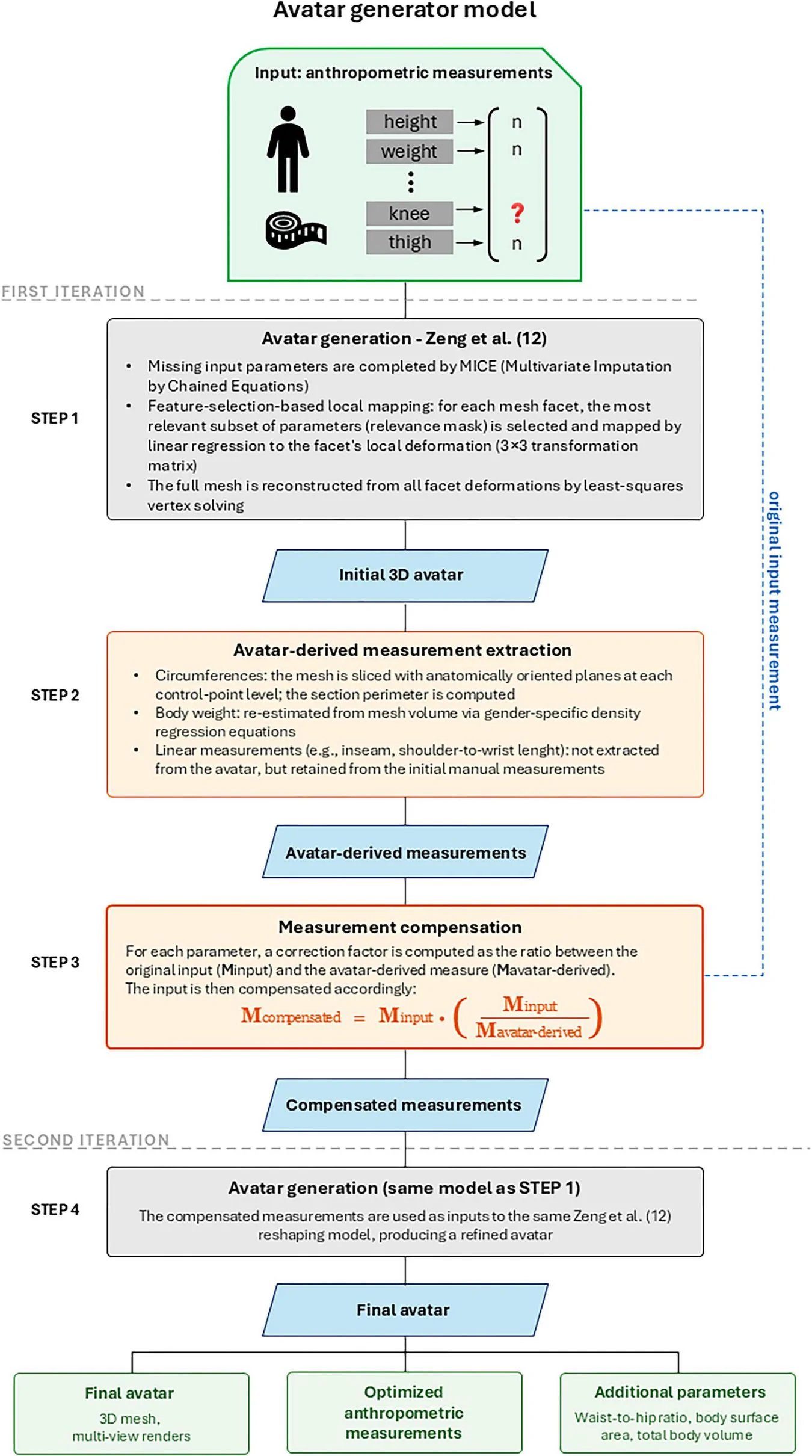
Step-by-step generation of the synthetic avatar, from manual input measurements to the final compensated avatar mesh.

### 2.6 Image processing

As shown in Figure 2 for a representative male, the raw mesh files obtained from both the digital and manual anthropometric assessments (panels A-B) were digitally registered and reposed (panels C-D) using Meshcapade (Meshcapade GmbH, Tübingen, Germany) (16) to standardize the vertices and pose (T-pose was adopted because it enables to remove posture-induced noise in ground-truth avatars) (17) and the following ground-truth and predicted measurements were extracted from the resultant avatars: neck base circumference, chest circumference, waist circumference, hip circumference, thigh circumference, inseam (Supplementary Table 2 and Supplementary Figure 3). In addition, the waist-hip ratio was calculated and body surface area and total body volume were estimated as, respectively, the sum of surface area of all triangles of the mesh and the enclosed space of the triangulated surface (by using the built-in volume property of the PyVista library) (18).

**FIGURE 2:**
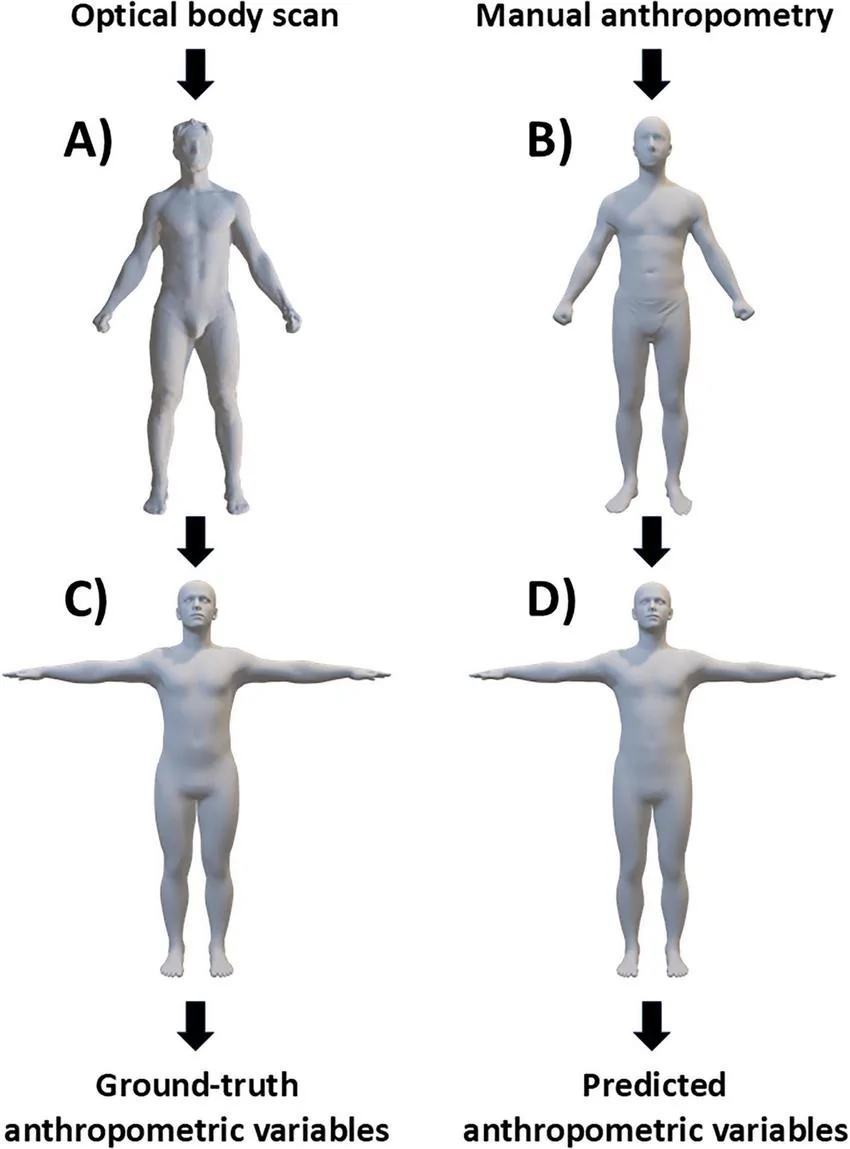
**(A)** Three-dimensional avatar obtained by optical body scan in a representative male subject (who gave his written consent for study participation and publication of anonymized images) and three-dimensional avatar predicted for the same subject from manual anthropometric measurements **(B)**. **(C,D)** Show the avatars digitally registered and reposed using Meshcapade (Meshcapade GmbH, Tübingen, Germany) (16) to obtain ground-truth and predicted anthropometric measurements.

### 2.7 Statistical analysis

Prior to statistical analyses, outliers of the ground-truth and predicted measurements were removed using the Grubbs’ outlier test (alpha = 0.05) (19) as a part of preprocessing quality control: the ground-truth and predicted neck circumferences of 1 female and the ground-truth and predicted thigh circumferences of 1 male and 1 female were identified as outliers and removed.

Comparison between ground-truth and predicted measurements in each of the two gender groups was performed using mean absolute errors (MAEs), normalized root-mean square errors (RMSEs), Bland-Altman analysis, and Pearson regression analysis (R^2^). Moreover, the Passing-Bablok regression analysis was also adopted for data comparison: this analysis was specifically designed to compare two data sets, given by two different methods, both affected by experimental errors (20, 21). If zero is not part of the 95% confidence interval of the intercept of the regression line, it may be concluded that systematic differences exist between the data acquired by the two methods. If 1 is not part of the 95% confidence interval of the slope of the regression line, it may be concluded that proportional differences exist between the compared data sets. For these conclusions to be valid, Passing and Bablok recommended a sample size of at least 50 subjects (20, 21): therefore, we grouped the data of the two genders to assess the differences between ground-truth and predicted measurements. The Passing-Bablok regression can only be applied if the data are well-fitted by a linear model: therefore, the Cusum test for linearity was also performed.

Data were reported as medians (1st–3rd quartile) or median (range) or mean (standard deviation: SD) as indicated. The threshold for statistical significance was set at *P* = 0.05. Statistical tests were performed using MedCalc v. 20.218 (MedCalc Software Ltd., Ostend, Belgium) and SPSS v. 20.0 (SPSS Inc., Chicago, IL, USA) software packages.

## 3 Results

### 3.1 Sample characteristics

Our sample included a total of 41 males [median (1st–3rd quartile) age: 32.5 (30.7–40.8) years] presenting a wide range of body mass index [median (range): 25.1 (19.2–39.0) kg/m^2^: 19 subjects had a normal weight, 11 were overweight, 11 were obese] and 42 females [median (1st–3rd quartile) age: 38.7 (28.2–51.6) years] presenting a wide range of body mass index [median (range): 22.3 (17.7–38.8) kg/m^2^: 1 subject was underweight, 31 subjects had a normal weight, 4 were overweight, 6 were obese].

### 3.2 Comparison between ground-truth and synthetic avatars

Figure 3 shows the heat maps of the differences (i.e., mean Euclidean distances) between corresponding points of the ground-truth and synthetic avatars reported on the average synthetic avatar of females (top panels) and males (bottom panels). The Euclidean distances were lower than 3 mm for the upper limbs and legs and were between 3 and 6 mm for the thighs. The chest and hip regions showed the greatest Euclidean distance values (between 12 and 15 mm). Briefly, ground-truth and synthetic avatars had similar visual appearances (as shown in Figure 2C, D for a representative male) and the differences were lower than 15 mm for all body regions in both females (Figure 3, top panels) and males (Figure 3, bottom panels).

**FIGURE 3:**
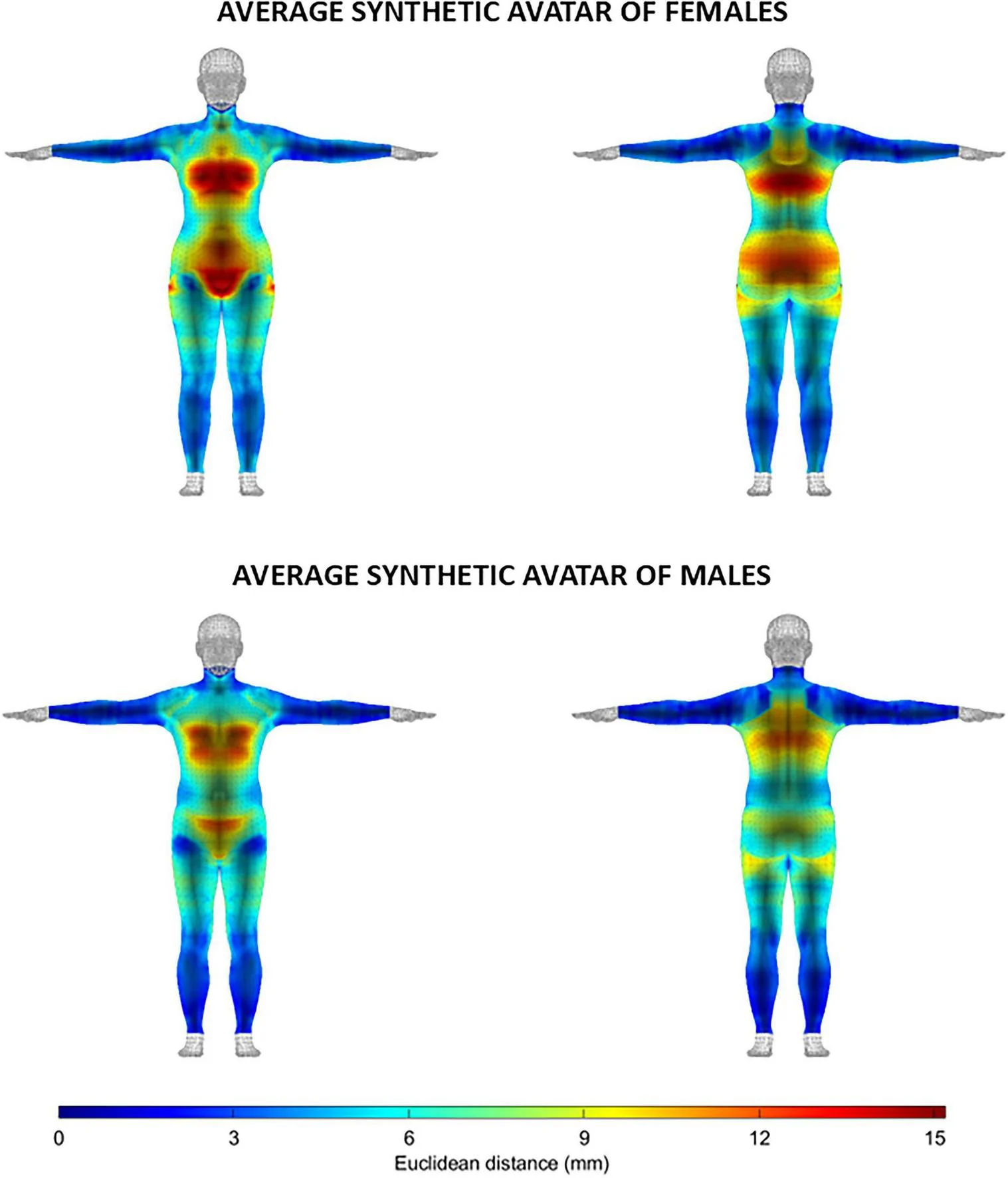
Heat maps of the differences (i.e., mean Euclidean distances) between corresponding points of the ground-truth and synthetic avatars reported on the average synthetic avatar of females (top panels: the frontal view is reported on the left, the dorsal view is reported on the right) and males (bottom panels): black indicates no differences, red indicates a difference of 12 mm.

### 3.3 Comparisons between anthropometric measurements obtained from ground-truth and synthetic avatars

The results of the comparison between ground-truth versus predicted anthropometric measurements are shown in Table 1 (for males) and Table 2 (for females) as medians (1st–3rd quartiles), MAEs, normalized RMSEs, Bland-Altman analysis-derived biases, and R^2^ values. MAEs, normalized RMSEs, and biases were all small in both males and females. The smallest MAEs values were observed in both genders for the waist-hip ratio and body surface area, while the highest MAEs value was observed for the chest circumference in males and for the inseam in females. The smallest value of the normalized RMSE was observed for the inseam in males and for the hip circumference in females, while the highest value was observed for the thigh circumference in males and for the neck base circumference in females. Consistently, the correlations between ground-truth and predicted measurements were all high (and statistically significant) with R^2^s ranging from 0.84 to 0.99 in males and from 0.77 to 0.99 in females. The lowest correlation was obtained for the thigh circumference in males and for the neck base circumference in females, while the highest correlation was obtained in both genders for the total body volume. As shown in Figure 4, significant positive correlations between ground-truth and predicted anthropometric measurements were obtained also for the grouped data of males and females: the R^2^ values ranged between 0.82 (for the thigh circumference) and 0.99 (for the total body volume). As shown in Table 3, the *P*-values obtained by the Cusum test for linearity (range of *P*-values between 0.26 and 1.0) indicated that the Passing-Bablok regression was applicable. The regression analyses showed systematic (the 95% confidence intervals of the regression intercepts did not include the 0 value) and proportional (the 95% confidence intervals of the regression slopes did not include the 1 value) differences between ground-truth and predicted measurements for some variables (neck base circumference, chest circumference, hip circumference, thigh circumference, inseam), while neither systematic nor proportional differences were observed for waist circumference, waist-hip ratio, body surface area, and total body volume.

**TABLE 1:** Comparison between anthropometric variables obtained from optical scan-derived avatars and from predicted avatars in 41 males.

Variables	Ground-truth measurement	Predicted measurement	MAE ( ± SD)	Normalized RMSE (%)	Bias	R^2^
Neck base circumference (cm)	42.6 (40.5–46.5)	42.6 (40.4–45.8)	0.7 (0.6)	2.1%	0.3	0.94
Chest circumference (cm)	107.6 (100.5–118.1)	103.6 (96.5–114.2)	4.1 (2.3)	4.4%	3.9	0.94
Waist circumference (cm)	90.9 (83.9–109.9)	90.9 (83.1–109.0)	1.5 (1.1)	2.1%	1.1	0.98
Hip circumference (cm)	104.3 (99.1–110.9)	103.5 (98.0–112.5)	1.4 (0.9)	1.6%	0.8	0.99
Waist–hip ratio	0.88 (0.84–0.94)	0.89 (0.85–0.94)	0.01 (0.01)	2.3%	0.003	0.96
Thigh circumference (cm)	65.0 (61.8–69.6)	63.2 (59.1–65.9)	2.6 (1.9)	4.9%	2.1	0.84
Inseam (cm)	78.5 (75.5–80.1)	78.3 (75.0–80.2)	1.1 (0.9)	1.5%	0.01	0.86
Body surface area (m^2^)	1.986 (1.864–2.101)	1.950 (1.864–2.101)	0.020 (0.04)	2.3%	0.024	0.97
Total body volume (l)	84.4 (75.0–100.7)	82.9 (75.0–97.9)	1.2 (2.1)	2.8%	1.2	0.99

Reported values are medians (1st–3rd quartile). RMSE: root mean square error.

**TABLE 2:** Comparison between anthropometric variables obtained from optical scan-derived avatars and from predicted avatars in 42 females.

Variables	Ground-truth measurement	Predicted measurement	MAE ( ± SD)	Normalized RMSE (%)	Bias	R^2^
Neck base circumference (cm)	37.1 (35.2–38.2)	35.7 (34.0–37.3)	1.5 (1.5)	5.7%	1.4	0.77
Chest circumference (cm)	93.1 (87.3–98.6)	92.2 (86.8–97.9)	1.8 (1.6)	2.7%	1.3	0.93
Waist circumference (cm)	78.3 (71.3–86.4)	79.6 (72.3–86.9)	1.8 (1.6)	3.1%	−1.2	0.97
Hip circumference (cm)	98.5 (95.2–105.9)	98.9 (96.4–109.1)	1.4 (1.2)	1.8%	−0.7	0.94
Waist-hip ratio	0.78 (0.76–0.82)	0.80 (0.76–0.83)	0.01 (0.01)	2.6%	−0.006	0.88
Thigh circumference (cm)	61.8 (59.1–67.2)	60.9 (58.8–64.3)	2.0 (1.8)	4.4%	0.9	0.78
Inseam (cm)	74.2 (71.7–75.7)	72.1 (69.7–73.5)	2.2 (0.9)	3.2%	2.2	0.91
Body surface area (m^2^)	1.647 (1.601–1.790)	1.646 (1.601–1.716)	0.025 (0.042)	3.0%	0.03	0.94
Total body volume (l)	63.2 (58.1–76.8)	63.1 (58.0–74.4)	0.9 (1.3)	2.5%	0.9	0.99

Reported values are medians (1st–3rd quartile). MAE, mean absolute error; RMSE, root mean square error.

**FIGURE 4:**
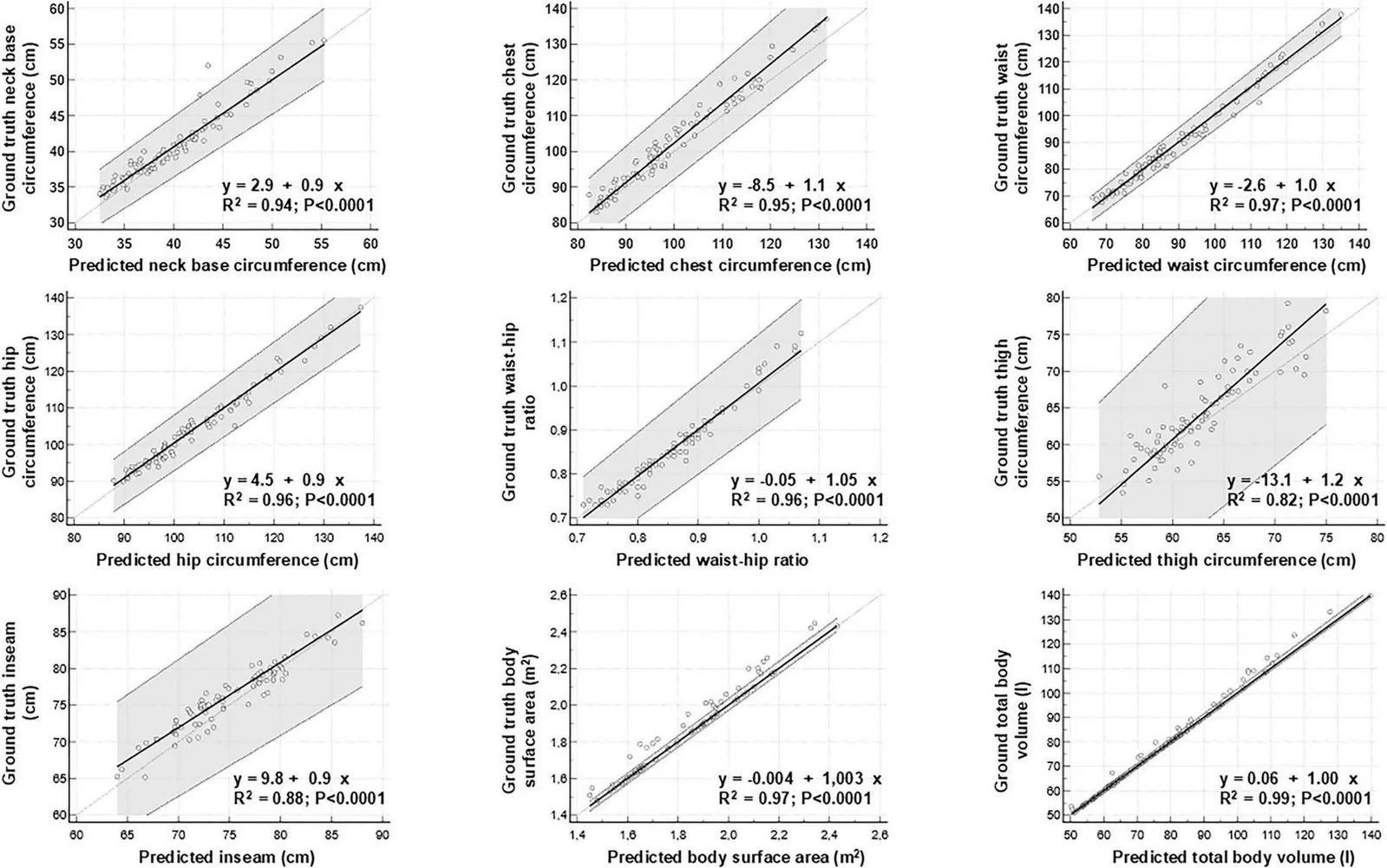
Relations between the ground-truth and predicted measurements investigated through the Passing-Bablok regression analysis. The regression plots include the line of identity and the generated regression line flanked by dotted lines depicting its confidence interval (shaded in grey) along with the R^2^ and *P*-value (obtained through the Pearson correlation).

**TABLE 3:** Results of the Passing-Bablok regression analyses.

NECK BASE CIRCUMFERENCE	
Linear model validity Cusum test for linearity	*P* = 0.16
Systematic differences	
• Intercept	2.92
• 95% confidence interval	0.96 to 5.20
Proportional differences	
• Slope	0.94
• 95% confidence interval	0.88 to 0.99
CHEST CIRCUMFERENCE	
Linear model validity Cusum test for linearity	*P* = 0.26
Systematic differences	
• Intercept	−8.51
• 95% confidence interval	−13.80 to −3.49
Proportional differences	
• Slope	1.11
• 95% confidence interval	1.06 to 1.16
WAIST CIRCUMFERENCE	
Linear model validity Cusum test for linearity	*P* = 0.57
Systematic differences	
• Intercept	−2.61
• 95% confidence interval	−5.15 to 0.10
Proportional differences	
• Slope	1.03
• 95% confidence interval	0.99 to 1.06
HIP CIRCUMFERENCE	
Linear model validity Cusum test for linearity	*P* = 0.40
Systematic differences	
• Intercept	4.55
• 95% confidence interval	0.67 to 8.47
Proportional differences	
• Slope	0.96
• 95% confidence interval	0.92 to 0.99
WAIST-HIP RATIO	
Linear model validity Cusum test for linearity	*P* = 0.57
Systematic differences	
• Intercept	−0.05
• 95% confidence interval	−0.10 to 0.00
Proportional differences	
• Slope	1.05
• 95% confidence interval	1.00 to 1.12
THIGH CIRCUMFERENCE	
Linear model validity Cusum test for linearity	*P* = 1.0
Systematic differences	
• Intercept	−13.14
• 95% confidence interval	−20.96 to −5.96
Proportional differences	
• Slope	1.23
• 95% confidence interval	1.12 to 1.36
INSEAM	
Linear model validity Cusum test for linearity	*P* = 0.76
Systematic differences	
• Intercept	9.78
• 95% confidence interval	4.48 to 14.16
Proportional differences	
• Slope	0.89
• 95% confidence interval	0.83 to 0.96
BODY SURFACE AREA	
Linear model validity Cusum test for linearity	*P* = 0.57
Systematic differences	
• Intercept	−0.004
• 95% confidence interval	−0.03 to 0.00
Proportional differences	
• Slope	1.00
• 95% confidence interval	1.00 to 1.02
TOTAL BODY VOLUME	
Linear model validity Cusum test for linearity	*P* = 0.57
Systematic differences	
• Intercept	0.06
• 95% confidence interval	−0.96 to 0.08
Proportional differences	
• Slope	1.00
• 95% confidence interval	−0.99 to 1.01

### 3.4 Comparisons between avatar-derived anthropometric measurements predicted through different models

Previously published models as well as our model can be used to create an avatar (and obtain anthropometric measurements) from a few input measurements (i.e., in the presence of missing data).

Figures 5, 6 show three-dimensional avatars of the participants in the Surmount 1 trial at the baseline and 72-week time point (i.e., before and after pharmacological intervention with tirzepatide) predicted from height, weight, and waist circumference (values are reported in the Supplementary Table 3). The top panel in both figures shows (front and side images of) the avatars predicted by Ramirez et al. (10) through the manifold regression model previously developed and validated by McCarthy et al. (9), while the bottom panel in both figures shows the avatars predicted through our model. Table 4 reports circumferences, body surface areas, and total body volumes obtained in males and females for the two time points (pre- and post-intervention: baseline and after 72 weeks) by Ramirez et al. (10) and by our model. Similar pre- and post-intervention measurements were obtained by the two different models for all variables, with the exception of the thigh circumferences: values obtained for the latter variable by Ramirez et al. (10) were higher both before and after the intervention compared to the values obtained by our model.

**FIGURE 5:**
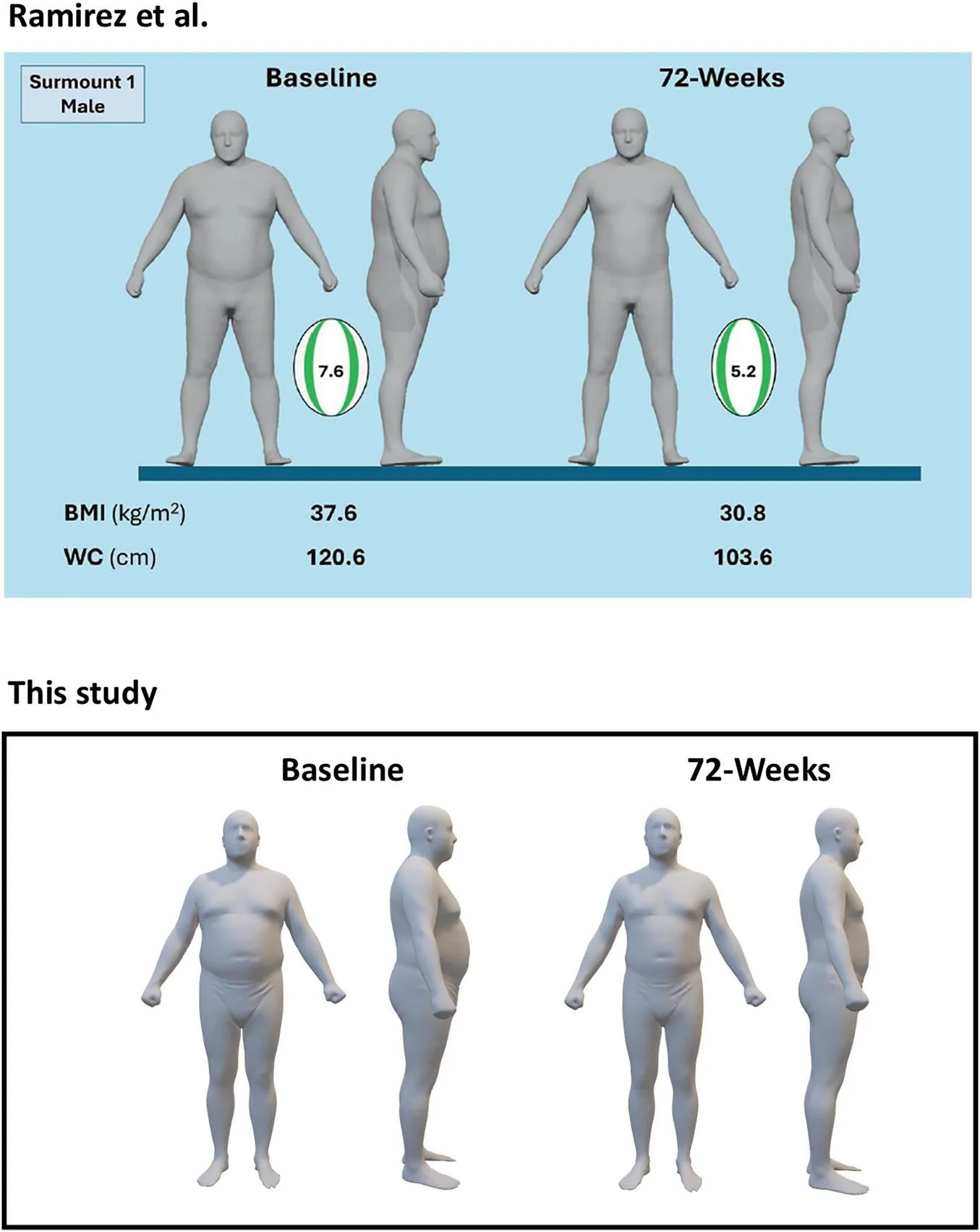
Three-dimensional avatars of the male participants in the Surmount 1 trial at the baseline and 72-week time point (i.e., before and after pharmacological intervention). The top panel shows (front and side images of) the avatars predicted by Ramirez et al. (10) through the manifold regression model previously developed by McCarthy et al. (9), while the bottom panel shows the avatars predicted through our model. BMI, body mass index: WC, waist circumference. The top panel has been reproduced from Ramirez et al. (10), licensed under CC BY-NC-ND 4.0.

**FIGURE 6:**
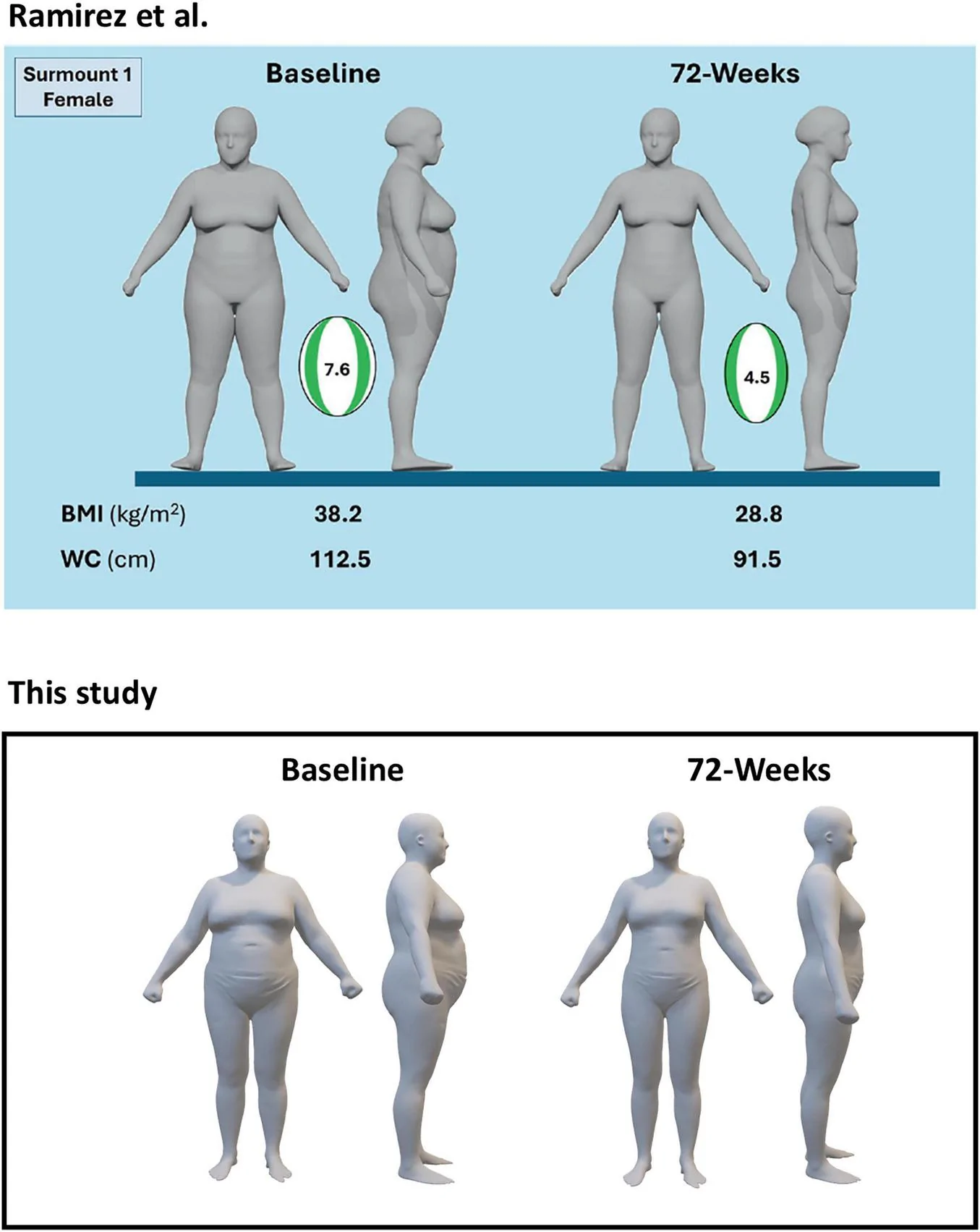
Three-dimensional avatars of the female participants in the Surmount 1 trial at the baseline and 72-week time point (i.e., before and after pharmacological intervention). The top panel shows (front and side images of) the avatars predicted by Ramirez et al. (10) through the manifold regression model previously developed by McCarthy et al. (9), while the bottom panel shows the avatars predicted through our model. BMI, body mass index; WC, waist circumference. The top panel has been reproduced from Ramirez et al. (10), licensed under CC BY-NC-ND 4.0.

**TABLE 4:** Baseline and 72-week characteristics of participants in the Surmount 1 trial obtained by Ramirez et al. (10) from the avatars predicted (from height, weight, and waist circumference values reported in the Supplementary table 3) through the manifold regression model previously developed by McCarthy et al. (9) and obtained for the avatars predicted through the model presented in this study.

Variables	Males		Females	
	Baseline	72 weeks	Baseline	72 weeks
Ramirez: Chest circumference (cm)	129.7	117.9	123.3	107.0
This study: Chest circumference (cm)	128.4	116.4	126.9	105.5
Ramirez: Hip circumference (cm)	125.8	112.0	128.7	114.3
This study: Hip circumference (cm)	116.9	108.5	121.6	108.2
Ramirez: Right mid-upper arm circumference	38.4	35.2	38.0	32.1
Ramirez: Left mid-upper arm circumference	38.5	35.7	38.2	31.9
This study: Mid-upper arm circumference	39.0	35.5	38.0	32.2
Ramirez: Right mid-thigh circumference	75.1	65.8	75.2	59.1
Ramirez: Left mid-thigh circumference	74.2	65.9	76.2	58.9
This study: Thigh circumference	65.2	60.0	66.7	61.2
Ramirez: Body surface area (m^2^)	2.23	2.05	1.99	1.73
This study: Body surface area (m^2^)	2.12	1.96	1.91	1.71
Ramirez: Total body volume (l)	125.0	101.9	105.9	79.2
This study: Total body volume (l)	114.3	94.5	101.0	76.7

## 4 Discussion

This study aimed to compare synthetic and ground-truth avatars (and predicted vs ground-truth anthropometric measurements) obtained, respectively, by a previously developed model applied to manual anthropometric measurements and by digital anthropometric assessments performed in healthy subjects and persons with obesity. The main results of this comparison can be summarized as follows: i) ground-truth and synthetic avatars had similar visual appearances; ii) the correlations between ground-truth and predicted measurements were all high and statistically significant; iii) no systematic and no proportional differences between ground-truth and predicted measurements were observed for waist circumference, waist-hip ratio, body surface area, and total body volume; iv) systematic and proportional differences between ground-truth and predicted measurements were observed for neck base circumference, chest circumference, hip circumference, thigh circumference, inseam; v) measurement comparisons between synthetic avatars predicted by our model and by the manifold regression model previously proposed by McCarthy et al. (9) showed similarities in body shapes and measurements obtained for the participants in the Surmount 1 trial.

The demonstration that synthetic avatars are accurate (although with some bias for some measurements) compared to ground-truth avatars obtained by digital anthropometry represents a validation (in an independent sample of subjects) of the model previously developed by Zeng et al. (12) that was adapted through an iterative recalculation step (based on the differences between measured and estimated body weight and circumferences) aimed to improve the coherence between anthropometric measurements and the visual representation of body shape. Possible explanations for the observed biases include differences between synthetic and ground-truth avatars in soft tissue deformation for some body regions and landmark identification inconsistency: further studies are required to refine the model in order to reduce the systematic and proportional biases that could impact on applications such as volumetric tracking, custom orthotic fitting, or metabolic risk stratification.

It remains also to be established in future studies which subset of anthropometric measurements is sufficient to generate accurate avatars, potentially allowing a reduction in the number of measurements required in the clinical practice.

Regardless of these future model improvements, it is worth highlighting that the generation of synthetic avatars reduces both procedural burden and cost associated with specialized equipment (that cannot be available in resource-limited environments such as remote and extreme settings) required to generate ground-truth avatars. Consistently, synthetic avatars were already used in clinical research for different applications. For example, several recent studies used synthetic avatars derived from anthropometric measurements obtained in epidemiological studies to describe the body shape changes (and to estimate the relative increase of the metabolic risk) during the last decades (6, 22, 23). In addition, the use of synthetic avatars can also be useful in clinical practice to provide patients with a tangible representation of their body shape: visualizing the pre-post intervention changes of the body shape and/or visualizing possible changes of the body shape as a future “self” can be used to support patient education and engagement in weight loss nutritional programs (10, 24). In fact, patients are inherently more interested in how they look to the human eye than in the quantitative details about their body size and composition (25). Furthermore, synthetic avatars can also be used to personalize and optimize the effectiveness of (dietary intervention combined with) physical activity programs, for example in virtual reality-based exercise protocols (26, 27). In fact, visualizing an actual “self” during motor observation and/or execution tasks delivered through virtual reality can promote brain plasticity mechanisms and strengthen key motor pathways (26, 27). However, the present model currently enables to generate only static avatars in a standardized A-pose: further developments of the model are therefore required to enable dynamic human avatar generation that is required for virtual reality applications.

### 4.1 Limitations

This study has a few limitations that warrant consideration. First, the CAESAR database (15) was adopted for the model development by Zeng et al. (12): although this dataset was very large, it included only adult subjects from the American and European populations. Therefore, the avatar generation from anthropometric measurements could be less accurate for subjects of other demographic groups (i.e., other ethnic groups or children and elderly subjects). Moreover, the CAESAR database included only healthy subjects: therefore, the prediction model could create an inaccurate avatar in presence of pathological conditions affecting body shape such as scoliosis, malnutrition, lymphoedema or lipoedema, severe obesity (for example in presence of thighs touching). Second, the model implies the use of only one anthropometric measurement for the different appendicular sites instead of two measurements (one for each side): therefore, the avatar generation could be less accurate in subjects presenting with congenital or acquired inter-limb circumference or length asymmetries. Future studies similar to the current investigations are required to explore avatar anthropometric prediction in non-normative populations. Third, ground-truth avatars (and relative anthropometric measurements) were obtained by one of the commercially available body scanners: thus, our validation is specific to the adopted model of 3-dimensional optical device.

## 5 Conclusion

This study showed that anthropometric measurements obtained from synthetic avatars are comparable (although with some bias for some measurements) to ground-truth avatars obtained by digital anthropometry. To promote the clinical use of digital anthropometry, the avatar generator model presented in this study was made available as a simple user interface-based calculator on the following web site: https://www.avatargenerator.unito.it/ (procedures for generating avatars are reported in Supplementary Table 4). Although several artificial intelligence-based avatar generators are freely available on the web, it is worth mentioning that our calculator is the only freely available tool that was developed from a large database of subjects and independently validated (through comparisons between synthetic and ground-truth avatars and derived measurements) in a separate population of healthy subjects and persons with obesity. We suggest that the systematic incorporation of avatars (and avatar-derived anthropometric measurements) into routine examinations of the patients can be useful for tailored clinical care to support patient education and engagement in weight loss nutritional programs.

## Data Availability

The raw data supporting the conclusions of this article will be made available by the authors, without undue reservation.

## Supplementary material

Supplementary Figure 1 Measurements obtained by manual anthropometry: neck circumference, shoulder width, upper limb length, mid-upper arm circumference, wrist circumference, chest circumference.

Supplementary Figure 2 Measurements obtained by manual anthropometry: natural waist circumference, belly button waist circumference, maximal hip circumference, thigh circumference, knee circumference, inseam.

Supplementary Figure 3 Ground-truth and synthetic avatars were digitally registered and reposed using Meshcapade (Meshcapade GmbH, Tübingen, Germany) (16) and the following measurements were extracted from the resultant avatars: neck base circumference, chest circumference, waist circumference, hip circumference, thigh circumference, inseam.
