# Supplementary material for: Avatar generator: a tool for prediction of three-dimensional humanoid avatars from manual anthropometric measurements
Source: Front Nutr. 2026 Sep 15;13:1906710. doi: 10.3389/fnut.2026.1906710 (PMC13621849; doi:10.3389/fnut.2026.1906710)

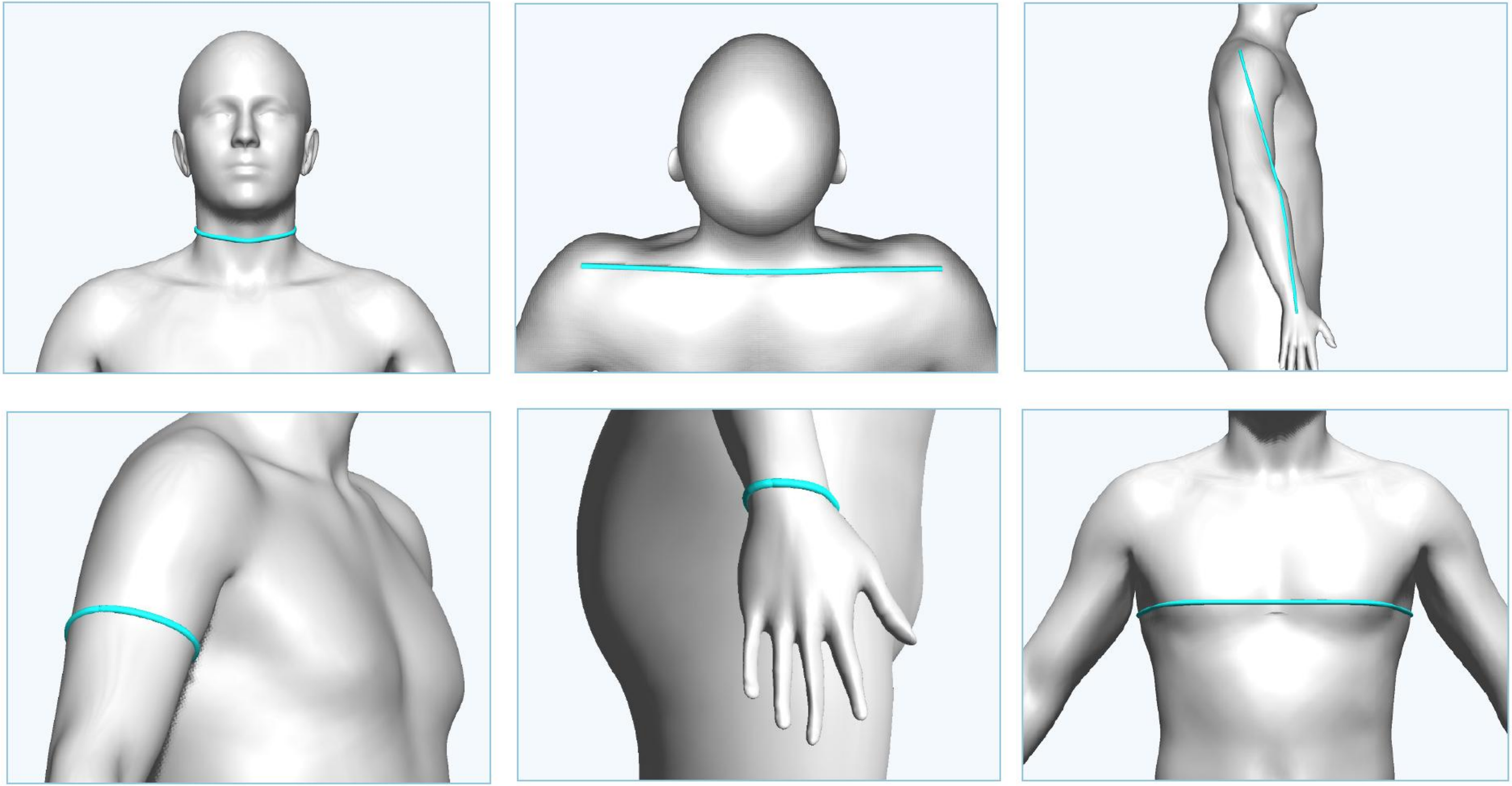

Supplement: Supplementary file 1 [file Image_1.pdf]
