# Supplementary material for: Avatar generator: a tool for prediction of three-dimensional humanoid avatars from manual anthropometric measurements
Source: Front Nutr. 2026 Sep 15;13:1906710. doi: 10.3389/fnut.2026.1906710 (PMC13621849; doi:10.3389/fnut.2026.1906710)
Supplement: Supplementary file 4 [file Table_1.pdf]

Supplementary Table 1

Measurement	Anatomical landmarks
Neck	Circumference of the neck mid-point
Shoulder width	Horizontal distance measured across the back from one shoulder to the other
Upper limb length	Distance between the most superior lateral point of acromion process (acromial landmark) and the lower and lateral border of styloid process of radius (radial landmark).
Mid-upper arm	Circumference of the upper arm measured at the mid-point between the shoulder and the elbow
Wrist	Circumference of the wrist measured distal to the styloid process of the radius and ulna.
Chest	Circumference of the chest measured at nipple level
Natural waist	Circumference of the narrowest part of the torso measured between the bottom of the ribcage and the top of the iliac crest
Belly button waist	Circumference of the waist measured at belly button
Maximal hip	Circumference of the widest point of the hips measured between crotch and hip bones
Thigh	Circumference of the thigh measured at its maximum horizontal near the top of the leg just below the gluteal fold
Knee	Circumference of the knee measured across the midpoint of the patella
Inseam	Vertical distance between the crotch and the floor measured along the inner side of the leg

Supplementary Table 1. Anatomical landmarks considered for the manual anthropometric assessment of circumferences and lengths.
