# Supplementary material for: Avatar generator: a tool for prediction of three-dimensional humanoid avatars from manual anthropometric measurements
Source: Front Nutr. 2026 Sep 15;13:1906710. doi: 10.3389/fnut.2026.1906710 (PMC13621849; doi:10.3389/fnut.2026.1906710)
Supplement: Supplementary file 5 [file Table_2.pdf]

Supplementary Table 2

Measurement	Measurement description
Neck base	The measurement of the circumference of the base of the neck is taken by measuring the distance around the neck at both<br><br>- the back neck point, at which the neck base line intersects with the anterior border of the trapezius muscle, and<br><br>- the front neck point, which is the crossing point of the line connecting the medial superior borders of the left and right clavicles with the front median line.
Chest	The chest measurement is the horizontal circumference taken at the level of the bust point, which is the foremost point of the bust.
Waist	The waist measurement refers to the horizontal circumference at waist level, situated at the midpoint between the lowest rib point and the highest point of the hip bone.
Hips	The hip measurement is the horizontal circumference of the body taken at the top hip level. This level is located midway between the waist level (which is the midpoint between the lowest rib point and the highest point of the hip bone at the side of the body) and the hip level (which is the level of the posterior projection at the back of the body, specifically the buttocks).
Thigh	The thigh girth is measured as the maximum horizontal circumference of the thigh, specifically below the natural crease or indentation known as the gluteal fold. The gluteal fold is the natural crease that forms at the point where the buttocks and the upper thigh meet.
Inseam	The inseam measurement is determined by observing the highest visible point at the junction between the right and left thighs from the back of the subject. It represents the vertical distance from this inside leg level down to the ground.

Supplementary Table 2. Measurements used on Meshcapade's avatar platform "Meshcapade Me".
