# Supplementary material for: Avatar generator: a tool for prediction of three-dimensional humanoid avatars from manual anthropometric measurements
Source: Front Nutr. 2026 Sep 15;13:1906710. doi: 10.3389/fnut.2026.1906710 (PMC13621849; doi:10.3389/fnut.2026.1906710)
Supplement: Supplementary file 6 [file Table_3.pdf]

Supplementary Table 3

VARIABLES	MALES		FEMALES	
	**Baseline**	**72 weeks**	**Baseline**	**72 weeks**
**Weight (kg)**	115.2	94.4	99.8	75.3
**Height (cm)**	175.0	175.0	161.6	161.6
**Waist circumference (cm)**	120.6	103.6	112.5	91.5

Supplementary Table 3. Baseline and 72-week characteristics of participants in the Surmount 1 trial [reproduced from Ramirez et al. (10)].
