# Supplementary material for: Avatar generator: a tool for prediction of three-dimensional humanoid avatars from manual anthropometric measurements
Source: Front Nutr. 2026 Sep 15;13:1906710. doi: 10.3389/fnut.2026.1906710 (PMC13621849; doi:10.3389/fnut.2026.1906710)
Supplement: Supplementary file 7 [file Table_4.pdf]

Supplementary Table 4

**1**	Open the website https://www.avatargenerator.unito.it/
**2**	Enter the required information (* indicates mandatory fields) in the Personal info section, including name and surname (or initials or ID code), evaluation date, gender (select male or female), weight, height
**3**	Enter the manual anthropometric measurements in the Optional measurement section
**4**	Click on the Generate Avatar button
**5**	Review the automatically generated avatar
**6**	Download the PDF report
**7**	Download the PNG files (front and back views)
**8**	Download the GIF file

Supplementary Table 4. Description of the procedures for generating avatars.
